# Gene-environment interactions and obesity: recent developments and future directions

**DOI:** 10.1186/1755-8794-8-S1-S2

**Published:** 2015-01-15

**Authors:** Tao Huang, Frank B Hu

**Affiliations:** 1 Department of Nutrition, Harvard School of Public Health, Boston, MA 02115, USA; 2 Department of Epidemiology, Harvard School of Public Health, Boston, MA 02115, USA; 3 Channing Division of Network Medicine, Department of Medicine, Brigham and Women’s Hospital and Harvard Medical School, Boston, MA 02115, USA

## Abstract

Obesity, a major public health concern, is a multifactorial disease caused by both environmental and genetic factors. Although recent genome-wide association studies have identified many loci related to obesity or body mass index, the identified variants explain only a small proportion of the heritability of obesity. Better understanding of the interplay between genetic and environmental factors is the basis for developing effective personalized obesity prevention and management strategies. This article reviews recent advances in identifying gene-environment interactions related to obesity and describes epidemiological designs and newly developed statistical approaches to characterizing and discovering gene-environment interactions on obesity risk.

## Introduction

Obesity has become a major public health concern. The number of overweight and obese adults has been estimated to be 1.35 billion and 573 million respectively by 2030 [1]. Obesity is associated with increased risk of chronic diseases and decreased health-related quality of life and overall life expectancy [2]. It is also associated with substantially elevated healthcare cost [3].

Obesity results from a complex interplay of many genetic factors and environmental factors [4-8]. Numerous epidemiological studies and clinical trials have examined the roles of lifestyle/dietary and genetic factors in the development of obesity. The body of evidence on gene-environment interaction (GEI) has also grown rapidly. However, preliminary results regarding GEI on obesity are for the most part inconclusive. The present review summarizes recent advances in identifying GEI related to obesity, and examines the newly developed approaches to testing GEI in the context of GWAS for obesity risk.

## Basic concepts

### a) Nutritional genomics

Nutritional genomics is an emerging field that may improve dietary guidelines for chronic disease prevention [9]. It covers both nutrigenomics and nutrigenetics. Nutrigenomics explores the effects of nutrients or other dietary factors on the gene expression, DNA methylation, proteome and metabolome [10], while nutrigenetics is aimed to elucidate whether genetic variations modify the relationships between dietary factors and risk of diseases [11]. Nutrigenetics has the potential to provide scientific evidence for personalized dietary recommendations based on the individual’s genetic makeup for weight control [9].

### b) Gene - environment interactions

In epidemiology, interaction is defined by estimating whether the degree of risk attributable to the joint effects of a genotype and an environmental factor on an outcome is greater or less than would be expected if these joint effects were additive [12]. Alternatively, GEI exists where the risk conveyed by specific genotype depends on one or more environmental exposure levels. This definition is quite helpful in the context of intervention studies where the environmental exposures can be intervened upon, such as diet and physical activity, to offset genetic risk [13-15]. Nutrigenetics is a special area of GEI research, where the environmental exposure is consumption of specific foods or nutrients. Looking from a different perspective, nutrigenetic studies also assess whether genetic factors modify the effects of specific dietary factors on diseases or related traits.

## Approaches to studying GEI

### a) Study designs for testing GEI

Over the past two decades, various study designs such as prospective cohort studies, case-control studies, case-only studies, randomized intervention trials, and twin studies have been used to test GEI [12]. Each design has its own advantages and disadvantages, and may be suitable for different situations.

#### Case-control studies

In population-based case-control studies, incident or prevalent cases in the studied population are ascertained over a certain time period, while the controls are randomly selected from the same source population. For example, a case-control design including 159 case subjects (BMI>30 kg/m^2^) and 154 controls (BMI<25 kg/m^2^) found that the *ADRB2* genotype modified the effect of carbohydrate consumption on obesity risk [16]. This finding suggested that high carbohydrate consumption was associated with an increased risk of obesity only among women with the Glu27 allele (OR 2.56, p=0.051). A Spanish case-control study reported that dietary saturated intake modified the effect of the *FTO* rs9939609 on risk of obesity among children and adolescents. The risk allele carriers consuming more than 12.6 % saturated fatty acids (of total energy) had an increased obesity risk compared with TT carriers [17], but the increased risk was not observed among those with lower saturated fat intake.

#### Case-only studies

Case-only studies can be used if the interest is limited to GEI, because the case-only design has the practical advantage that there is no need to collect control samples. This design is based on the assumption that genotypes and environmental exposures are independent of each other, so that the exposures should not differ among different genotypes. The case-only design is more efficient than case-control design, but the independence assumption may not hold. In addition, the design is subject to bias and confounding, especially if there is exposure misclassification [18]. For example, a case-only study among 549 adult obese women observed an interaction between fiber intake and the -514 C>T polymorphism of the *LIPC* gene (p for interaction=0.01). Similarly, the -11377G>C polymorphism of the *ADIPOQ* gene and the -681 C>G polymorphism of the *PPARG3* gene were found to modify the association of dietary fat intake and obesity (all p for interaction<0.05) [19].

#### Cohort studies

The classic prospective cohort study follows subjects over time, comparing the outcome of interest in individuals who are exposed or not exposed at baseline [5]. Because exposure is assessed before the outcome, the cohort design is less susceptible to selection bias and differential recall bias between cases and noncases when compared to a case-control design. However, cohort studies of chronic conditions with low incidence are expensive, and require large sample size and long follow-up. A nested case-control study within a large prospective cohort can improve efficiency and reduce cost [20]. In recent years, several cohort studies have investigated the GEI on obesity. For example, Qi et al. calculated weighted genetic risk score (GRS) on the basis of 32 BMI variants and demonstrated that the genetic association with adiposity was stronger among participants with higher intake of sugar-sweetened beverages than among those with lower intake in the Nurses' Health Study (NHS) and the Health Professionals Follow-up Study (HPFS) cohorts, and these findings were replicated in the Women's Genome Health Study (WGHS) cohort [8]. A similar interaction between regular consumption of fried food and GRS in relation to obesity was observed among these three cohorts [6]. In the combined analysis, the differences in BMI per 10 risk alleles were 1.1 (SE 0.2), 1.6 (SE 0.3), and 2.2 (SE 0.6) for fried food consumption less than once, one to three times, and four or more times a week (p<0.001 for interaction). These findings suggested that the genetic association with adiposity was strengthened with higher consumption of fried foods. Furthermore, it was documented the genetic association with BMI was strengthened with increased hours of TV watching in 7740 women and 4564 men from the NHS and HPFS. In contrast, the genetic association with BMI was weakened with increased levels of physical activity. These findings suggest that sedentary lifestyle may enhance the predisposition to elevated adiposity, whereas greater leisure time physical activity may mitigate the genetic association [21].

#### Clinical trials

Randomized controlled trial (RCT) is widely considered to be the most reliable design because of the randomized allocation of the exposures. However, RCT is often infeasible to test the long-term effects of dietary exposures on obesity or obesity-related chronic diseases due to cost and logistic considerations. Several randomized dietary intervention trials of weight loss have been analyzed to provide unique insights into individualized dietary response to weight loss diets based on specific genetic variants (Table 1). The Preventing Overweight Using Novel Dietary Strategies Trial is the largest and longest-term (2-years) randomized intervention trial comparing the effects of four weight-loss diets of varying macronutrient compositions [22]. The results from this trial showed that individuals carrying the C allele of the branched-chain amino acid/aromatic amino acid ratio-associated variant rs1440581 might benefit less in weight loss than those without this allele when undertaking an energy-restricted high-fat diet [23]. For *FTO* variant rs1558902, a high-protein diet was found to facilitate weight loss and improvement of body composition in individuals with the risk allele of the *FTO* variant rs1558902, but not in other genotypes [24]. Several other intervention studies also demonstrated gene-diet interaction on obesity (**Table**1). For example, Alsaleh et al found that higher consumption of n-3 polyunsaturated fatty acids modified the effects of *ADIPOQ* rs2241766 on risk of obesity [25]. Improvement in metabolic markers secondary to weight loss was greater in *FTO* rs9939609 A allele carriers with a low-fat hypocaloric diet [26]. The *FAAH* rs324420 AA/AC was not associated with weight loss in a 1-year lifestyle intervention for obese children and adolescents [27]. These results need to be validated in further studies.

**Table 1:** Summary of selected intervention and cohort studies on gene-diet interactions during the past two years

Author	Study design	Genetic markers	Main findings
Qi et al. 2011[42]	Two years, intervention, n=738	*IRS* rs2943641	*IRS1* genetic variants modified effects of dietary carbohydrate on weight loss
Mattei et al. 2012 [43]	Two years, intervention, n=591	*TCF7L2* rs7903146	Dietary fat intake modified effect of *TCF7L2* genotype on changes in BMI, total fat mass, and trunk fat mass
Qi et al. 2012 [44]	Two years, intervention, n=737	*GIPR* rs2287019	Dietary carbohydrate modified *GIPR* genotype effects on changes in body weight
Xu, et al. 2013 [23]	Two years, intervention, n=734	*PPM1K* rs1440581	Dietary fat modified genetic effects on changes in weight
Alsaleh et al, 2013 [25]	One year, intervention, n=367	*ADIPOQ* rs2241766	A diet high in n-3 polyunsaturated fatty acids modified the effects of rs2241766 on risk of obesity
Knoll et al 2012 [27]	One year, intervention, n=453	*FAAH* rs324420	The *FAAH* rs324420 AA/AC is not associated with weight loss in a 1-year lifestyle intervention for obese children and adolescents
de Luis et al, 2013 [26]	Three months intervention, n=305	*FTO* rs9939609	Metabolic improvement secondary to weight loss was better in A carriers with a low fat hypocaloric diet
Lai et al 2013 [45]	Four weeks intervention, n=88	*Visfatin* rs4730153	*Visfatin* rs4730153 homozygous GG Genotype may affect glucose and lipid metabolism in obese children and adolescents by reducing total triglyceride level and increasing insulin sensitivity to exercise
Qi et al 2012 [8]	Cohorts (NHS, HPFS, WGHS)	BMI-GRS	The genetic association with adiposity was stronger among participants with higher intake of sugar-sweetened beverages than among those with lower intake.
Qi et al 2012 [21]	Cohorts (NHS, HPFS)	BMI-GRS	Sedentary lifestyle may accentuate the predisposition to elevated adiposity, whereas greater leisure time physical activity may attenuate the genetic association.
Qi et al 2014 [6]	Cohorts (NHS, HPFS, WGHS)	BMI-GRS	Participants in the highest risk groups for both fried food and GRS had the highest BMI overall. Eating fried food more than four times a week had twice the effect on BMI for those in the highest third of GRS than those in the lowest third.

GRS: genetic risk score, NHS: the Nurses' Health Study, HPFS: the Health Professionals Follow-up Study, WGHS, the Women's Genome Health Study.

The GRS was calculated on the basis of 32 established BMI-associated variants.

### b) Evolving Approaches to GEI: GWEI

The GWAS approach has made impressive progress in identifying common obesity genetic variants. However, GWAS analysis of main effects only might miss important genetic variants restricted to exposure subgroups of the population. Several approaches to assessing genome-wide environment interaction (GWEI) have been developed recently. These approaches also have the potential to identify novel SNPs that are not detected in genome wide scan. However, no study has reported the GWEI for obesity. In this section, we summarized the newly developed methods for GWEI that has the potential to detect GEI on obesity (**Table**2):

**Table 2:** Examples of newly developed genome-level approaches to GWEI.

Author	Year	Methods
Kraft et al	2007	Joint test of marginal effects of SNPs and G x E [34].
Murcray et al.	2009	Two-step analysis of GWAS data [28]
Paré et al	2010	Variance prioritization approach[37]
Wei et al.	2012	SNP, gene, and pathway based GWAS analysis[30]
Hsu et al.	2012	Cocktail methods [33]
Gauderman et al.	2013	Revised two-step screening and testing method (EDG×E) [29].
Jiao et al.	2013	SBERIA: Set-Based Gene-Environment Interaction Test [38].

#### 1) 2-step analysis

The 2-step approach incorporates a preliminary screening step to efficiently use all available information in the data [28]. In the first step (screening test), for each of the SNPs, a likelihood ratio test of association between genetic variant and environment was performed using a logistic model. The second step uses an unbiased traditional GEI test of the SNPs that passed the screening step to ensure an overall valid procedure. It was demonstrated that two-step approach reduced the number of SNPs tested for interactions and substantially improved the power of GWEI. Recently, an improved two-step screening and testing method (the screening step included exposure-genotype and disease-genotype information; EDG×E) was proposed. A software program which implements this new method and other GWEI approaches is now available (G×E scan, http://biostats.usc.edu/software) [29].

#### 2) Gene- or pathway-based approaches

Both gene-and pathway-based analytic approaches have been used to integrate prior biological knowledge into association and interaction analyses [30], by combining associations of genetic variants in the same gene or biological pathway. Therefore, it could enhance statistical power and also provide insights into biological mechanisms. Several recent studies have shown that gene-based and pathway-based approaches to GEI in the context of GWAS could facilitate the mining of functional information that is complementary to traditional agnostic GWAS analysis [31]. Wei et al. conducted a GWEI to identify gene-asbestos interaction in lung cancer risk at levels of SNPs, genes, and pathways, using Texas lung cancer GWAS dataset, and found that pathway-based analyses had more power than SNP- or gene-based analyses [32].

#### 3) A module-based cocktail approach

Hsu et al. proposed a module-based approach to integrating various methods (such as the correlation screening and marginal association screening) that exploits each method’s most appealing aspects [33]. Three modules were included in this approach: 1) a screening module for prioritizing SNP; 2) a multiple comparison module for testing GEI; and 3) a GEI testing module. They combined all three of these modules and developed two novel “cocktail” methods. It was demonstrated that the proposed cocktail methods did not inflate the type I error and had enhanced power under a wide range of situations [33]. This modular approach is computationally straightforward.

#### 4) A joint test of marginal associations and GEI

Kraftet al. proposed a joint test of marginal association and GEI [34], using a likelihood ratio test. The joint test was found to have greater power than the marginal test when the genetic association was confined to an exposure subgroup or the GEI test when the genetic association was detected in both exposed and non-exposed groups [34]. Several studies have demonstrated enhanced power for large-scale association studies where the true underlying GEI model is unknown [35,36].

#### 5) Variance prioritization approach

Pare et al. proposed a novel approach to prioritize SNPs for testing the gene-gene and gene-environment interactions for quantitative traits [37]. In this approach, the variance of a quantitative trait by genotypes in the presence of an interaction effect was calculated, and then Levene's test was used to test if subgroup samples have equal variances. Pare et al. further applied the variance prioritization approach in the Women's Genome Health Study and identified several novel interactions, including the interactions between the *LEPR* rs12753193 and BMI on C-reactive protein levels, between the *ICAM1* rs1799969 and smoking on intercellular adhesion molecule 1 (ICAM-1 ) levels [37]. Given the limited number of SNPs that are eventually tested for interactions, this approach has enhanced power over traditional methods.

#### 6) A set-based gene environment interaction test (SBERIA)

Jiao et al. proposed a set-based gene environment interaction test (SBERIA) to explore the GEI using case-control data [38]. SBERIA first selects markers with relatively strong correlation signals, and then a weighted sum of the selected marker interaction terms is computed, where the weight corresponds to the magnitude and direction of the correlations among the markers. SBERIA was applied to GWAS data of 10,729 colorectal cancer cases and 13,328 controls and the study identified several significant interactions of known susceptibility loci with smoking on colorectal cancer [38]. One advantage of SBERIA is that could increase the statistical power by aggregating correlated SNPs within a marker set and thus reduce the multiple testing problems.

## Continued challenges

Despite some progress in characterizing GEI underlying obesity, many challenges remain. First, many inconsistencies and significant findings need replication or more detailed follow-up. Publication bias may have contributed to the absence of replication reports. Therefore, it is critical for researchers to conduct replication studies and to publish both positive and negative results [39]. Second, inadequate statistical power due to modest sample sizes and measurement errors for environmental factors continue to be major factors limiting progress in the field [39]. Environmental exposures such as diet and exercise are often difficult to measure in free-living populations. Simulation studies have demonstrated that in GEI studies, even a modest amount of measurement errors in assessing environmental exposure can result in a substantial reduction in statistical power to detect an interaction [40].

## Future perspective of GEI on obesity

There has been considerable progress in our understanding of the role of both genetic and environment factors in the development of obesity. Findings to date indicate that behavioral changes such as improving diet and physical activity can substantially offset obesogenic effects of risk alleles, which has much broader clinical and public health implications. In the near future, individuals may be able to obtain their comprehensive genetic information and thus a knowledge of their genetic predisposition to obesity and other chronic diseases. Nutritional genetics studies have made slow but steady progress in examining gene and dietary intervention interactions for weight loss and maintenance [8,23,24,41], but there are still many challenges. Continued progress will depend on appropriate study design; more accurately measured environmental factors, and very large sample size. Further investment in studies of GEI for obesity holds promise on several grounds [39]. First, GEI studies may help us better understand disease mechanisms by providing biological insight into the function of novel obesity loci and pathways and interplays between the genes and environment. Second, GEI investigation may identify high-risk individuals for more efficient and targeted diet and/or lifestyle interventions. Finally, the integrating of genomics with other “omics” such as transcriptomics, proteomics, and metabolomics can provide greater insights into how diet and lifestyle alter the expression or ‘manifestation’ of our genomes and the interplays between genes and environments on obesity development and progression. This approach, termed “systems epidemiology” [39], has tremendous potential to advance our understanding of obesity etiology and to help achieve the goal of personalized nutrition for obesity prevention and management.

## Abbreviations

BMI: body mass index; GEI: gene-environment interaction; GWAS: genome wide association study; GWEI: genome-wide environment interaction; SNP: single-nucleotide polymorphism; GRS: genetic risk score

## Competing interests

The authors declare that they have no competing interests.

## Authors’ contributions

FH conceived the idea for the review. TH drafted the manuscript. Both authors read and approved the final manuscript.

## Funding source

This article was funded by the National Institutes of Health (NIH) grant DK58845.

## Declarations

This article has been published as part of *BMC Medical Genomics* Volume 8 Supplement 1, 2015: Selected articles from the 2nd International Genomic Medical Conference (IGMC 2013): Medical Genomics. The full contents of the supplement are available online at http://www.biomedcentral.com/bmcmedgenomics/supplements/8/S1
